# A New Application of Electricity

**Published:** 1864-05

**Authors:** 


A New Application of Electricity.—*To the Editor of the American Medical Times.*

Sir:—The utilization of this mysterious principle continues to occupy the earnest thoughts of subtle and ingenious minds. The discovery of its relationship to magnetism astonished the world scarcely less than that of its identity with lightning; and its application to telegraphy, the most valuable of all, seemed to put the climax to its utility. But new modes of making it subservient to the business and comforts of man are still more sought for, and one which was incidentally brought to our attention during a recent visit to Philadelphia, so struck our fancy by its beauty, usefulness, and convenience, that we were induced to pay a visit to the establishment where the idea found development and practice, and where, although a stranger, we found a welcome, and were politely shown the whole matter.

For two years past Mr. Robert Cornelius, of that city (the head of the large establishment of Cornelius and Baker), has been devoting his time to perfecting an arrangement for lighting gas as it issues from the ordinary bracket or chandelier, by the electric spark. The method of accomplishing this was apparent to his mind very early, but to bring the apparatus to such perfection as to insure its certain operation at all times and seasons, and under all circumstances and conditions of the weather, was the nice point upon which he has labored with great assiduity and ingenuity, until he has accomplished the desideratum in a manner which may be regarded as nearly, if not quite, infallible. The little apparatus for the purpose forms an additional ornament to each gas bracket or chandelier, and is ever ready for action, so that matches and torches may be wholly dispensed with, wherever it is introduced.

By a very gentle friction of two surfaces, a delicate spark of electricity is generated at the moment of the escape of the gas from the burner, which is instantly ignited. The apparatus consists, in the case of an ordinary bracket, of a small brass conical cup, with an inside lining of lamb's wool and silk, into which is loosely fitted a plug of hard rubber, surmounted by a knob or handle. The slight friction caused by simply lifting this plug from its bed in the cup, generates electricity sufficient for the purpose. This is conducted by a delicate copper chain or wire, covered with silk, to the orifice of the burner, where it is discharged from a platinum point through the jet of gas, and instantly ignites it.

Nothing can be more simple and beautiful, while the cup and chain are a decidedly ornamental addition to the bracket.

In the case of a chandelier with five burners, which we saw, the positive and negative surfaces were arranged in the form of circular disks, about six inches in diameter, lying horizontally in contact with each other. The act of separating these, by slightly raising the upper one, produces a spark which is transmitted to each burner by a separate conductor, and ignites all the jets of gas simultaneously. This action of separating the disks, as well as the "lettin on" of the current of gas, is performed by a very neat and ingenious contrivance, a single movement of a stopper being sufficient. It is so arranged also that any one of the five jets may be ignited independently of the others.

From the day when Franklin "eripuit cœlo fulmen," to that when Morse united the ends of the earth in an instantaneous bond, scarcely has there been seen a more pleasing and beautiful adaptation of electricity to human comfort and convenience. Though not yet introduced to the business public, permission was accorded us to present it to the readers of the *Medical Times*, and though our description, without a drawing, must necessarily be imperfect, we trust it is sufficiently clear as it regards the principle of its operation. In our judgment this beautiful invention, which will be regarded as a philosophic toy, as well as a household convenience, will soon be esteemed as indispensable as the sewing machine.

The glory of the friction match will then be dimmed, as surely as it extinguished that of the flint and steel and tinder-box. With sulphur and phosphorus dispensed with in our households, we shall hear no more of children mistaking matches for candies, and dying in consequence. All honor, we say, to the philosophic and patient genius of the American artist.

